# Navigating Careers in Ayurveda: A Comprehensive Needs Assessment for Introducing a Career Guidance Module

**DOI:** 10.7759/cureus.60519

**Published:** 2024-05-17

**Authors:** Shweta Telang-Chaudhari, Gaurang Baxi

**Affiliations:** 1 Department of AYUSH (Ayurveda, Yoga & Naturopathy, Unani, Siddha, and Homeopathy), Maharashtra University of Health Sciences, Nashik, Nashik, IND; 2 Department of Cardiorespiratory Physiotherapy, Dr. D. Y. Patil College of Physiotherapy, Dr. D. Y. Patil Vidyapeeth, Pune, Pune, IND

**Keywords:** guidance and counselling, internship training, ayurveda education, ayurveda curriculum, career option, career decisions

## Abstract

Introduction: The career landscape in Ayurveda is diverse and multifaceted. Many career opportunities are now being available for Ayurveda in many fields, e.g. cosmeceuticals, neutraceuticals, herbal pharmaceuticals, preventive healthcare, lifestyle and wellness and clinical research. However, an interactive platform is lacking to introduce recent Ayurveda graduates to emerging career opportunities. A dedicated career guidance module can help address these aspirations by providing insights into various career paths and potential career trajectories. This Needs Assessment Survey was conducted among different stakeholders within Ayurveda to find out the perceived need for introducing a career guidance module for interns.

Methodology: After institutional sub-ethics committee approval, a peer-validated needs assessment questionnaire for a career guidance module was developed and administered to faculty, clinicians, industry entrepreneurs, post-graduate residents, fresh graduates and interns within Maharashtra. Snowball sampling through WhatsApp was used to collect responses.

Results: A total of 102 responses were received. Fifty-eight per cent of respondents were females. An almost equal number of faculty, interns and fresh graduates responded. In carer preferences, post-graduation followed by clinical practice were two responses that received maximum votes. An academic career was least preferred. The majority of faculty and students felt that sufficient career options were available for Ayurveda graduates. Private practitioners and entrepreneurs felt otherwise. For updated information on career opportunities, interns rely on the internet over peers and local contacts. Guidance from colleges is minimal.

Conclusion: Internship is an important time in career decision-making. Internship experiences are likely to influence the opinions of fresh graduates regarding their desired career paths. Almost all survey participants agreed on an urgent need to have career guidance in Ayurveda. This should focus on employment, enhancing communication skills, professional ethics and leadership skills along with clinical expertise. The pursuit of a career in Ayurvedic research, Ayurvedic drug development, Ayurvedic manufacturing, Ayurvedic tourism, etc. lacks a clear career path. Most interns are unfamiliar with these uncharted career paths. The Health Universities, colleges and eminent experienced alumni of Ayurveda colleges can form an intensive network to guide and support students in making an appropriate choice of a career in Ayurveda.

## Introduction

Ayurveda, as an ancient system of medicine, is experiencing a resurgence in popularity worldwide. With this renewed interest, there's a growing number of individuals seeking education and careers in Ayurvedic practices. A career is not just a job or an employment to earn a livelihood. A job or a profession can be called as a career only when it has an element or opportunity for progress and advancement [1].

The career landscape in Ayurveda is diverse and multifaceted. However, navigating this landscape can be challenging for students and practitioners due to its complexity. With increasing globalization, Ayurveda graduates need to be competent and vigilant about the changing global scenario. A strong foundation in medical education, enabling students of their capabilities and inclinations, will help them in making an "informed" career choice of what they wish to achieve professionally. Although graduating medical students bring a wealth of medical knowledge and experience to the workplace, little is known about how well-prepared they are for the shift to practising medicine in a variety of settings [2].

Ayurveda is a growing healthcare Industry. Many career opportunities are now being available for Ayurveda in many fields, e.g. cosmeceuticals, neutraceuticals, herbal pharmaceuticals, preventive healthcare, lifestyle and wellness industry, clinical research, etc. However, we lack an interactive platform or suitable mechanism to introduce recent graduates to the Ayurvedic health sector's emerging career opportunities. Ayurveda education should be directed towards addressing issues of the community. Though there is an increased societal and industrial interest in Ayurveda, there is no present system to make our students interact directly with the world they would eventually step into. The mutual symbiotic existence of the Ayurveda fraternity, industry and the community needs to be strengthened with the establishment of a connecting network for the exchange of interaction.

There is little career counselling during medical school, and graduates believe this needs to change [3]. Despite the increasing interest in Ayurveda, a structured guidance module tailored to the specific career paths within the field is lacking. Students and practitioners may struggle to find relevant information and support to make informed career decisions. Many individuals entering the field of Ayurveda have diverse career aspirations and goals. A dedicated career guidance module can help address these aspirations by providing insights into various career paths, educational requirements, skill development opportunities, and potential career trajectories. Thus, this Needs Assessment Survey was conducted among Ayurveda interns to find out whether there is a perceived need for introducing a career guidance module for them. The evolving needs and aspirations of graduates entering and practising within the Ayurveda field were explored.

## Materials and methods

Ethical approval was taken from the Institutional Sub-ethics Committee. A Needs Assessment Questionnaire was meticulously designed to gather comprehensive data on the career needs, challenges, and aspirations of individuals within the Ayurveda community. It began with a thorough review of existing literature on career guidance in Ayurveda, ensuring that the questions were relevant and grounded in existing research. The questionnaire was structured to cover various aspects such as demographic details, educational background, career opportunities and preferences, sources of gathering information, perceived challenges, desired support mechanisms, and suggestions for establishing a structured career guidance training module. Before administration, the questionnaire underwent a peer validation process to ensure its validity and reliability. A panel of subject matter experts, including academicians, practitioners, and researchers in Ayurveda, reviewed the questionnaire for clarity, relevance, and comprehensiveness. Feedback from the experts was carefully considered and incorporated to refine the questionnaire, ensuring that it accurately captured the intended information and addressed the research objectives. The development, peer validation, and administration of the questionnaire followed a rigorous approach to data collection, aimed at capturing the diverse perspectives and experiences within the Ayurveda community regarding career guidance needs and preferences.

A Google form of the questionnaire was circulated through WhatsApp targetting different groups within the Ayurveda community within Maharashtra, including interns, fresh graduates, teachers from recognized Ayurveda colleges, and practising Ayurveda clinicians. Snowball sampling through WhatsApp was used to collect maximum responses from the Ayurveda community including faculty, clinicians, industry entrepreneurs, PG residents, fresh graduates and interns. Participants were asked to share the questionnaire among their known colleagues. They were provided with clear instructions on how to complete the questionnaire and were assured of the confidentiality and anonymity of their responses. Efforts were made to maximize the response rate through WhatsApp reminders, ensuring a representative sample and enhancing the validity of the findings. This process helped enhance the validity of the study findings, enabling informed decision-making and effective implementation of career guidance initiatives in Ayurveda.

## Results

**Figure 1:**
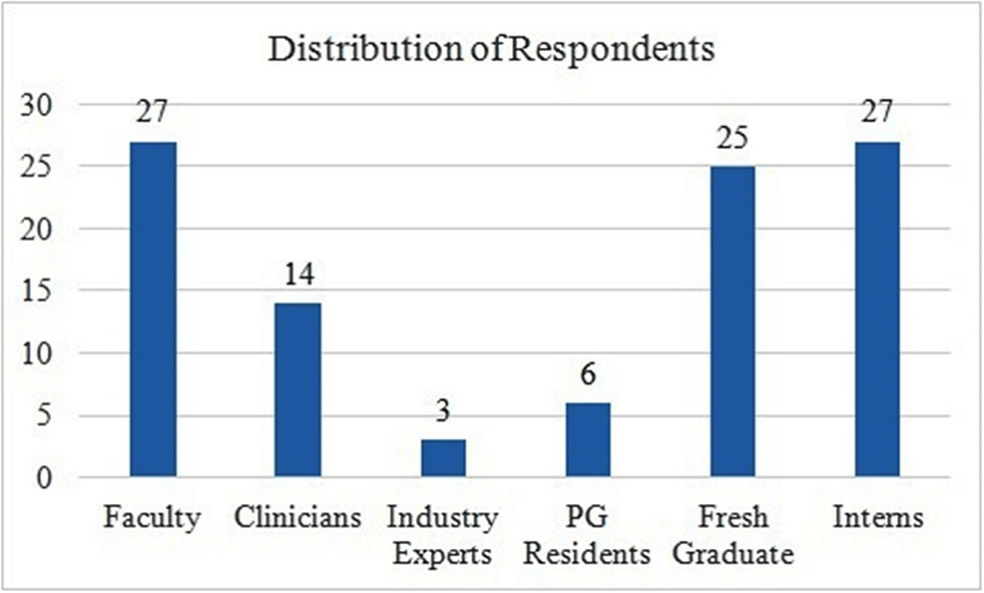
Participant distribution Total number of respondents n = 102. PG: postgraduates

Data collected was analysed using descriptive statistics and expressed as percentages. A total of 102 responses were received. Fifty-eight per cent (n=59) of respondents were females. An almost equal number of faculty, interns and fresh graduates responded to the questionnaire (Figure 1).

**Figure 2:**
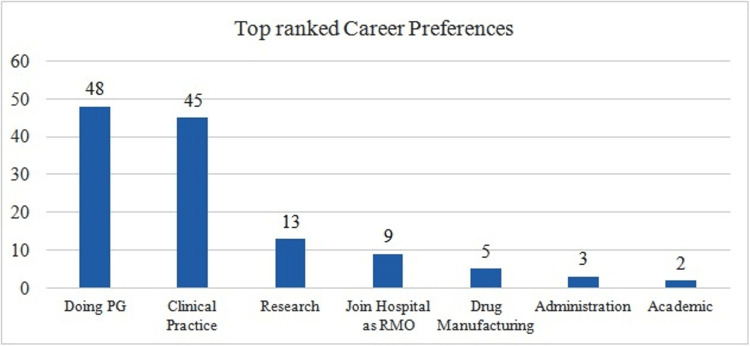
Top-ranked career preferences Total number of respondents = 102. RMO: Resident Medical Officer; PG: postgraduates

Going for post-graduation (n=48) followed by clinical practice (n=45) were the two responses which received maximum responses. Surprisingly, an academic career (n=2) was the least preferred career option (Figure 2).

**Figure 3:**
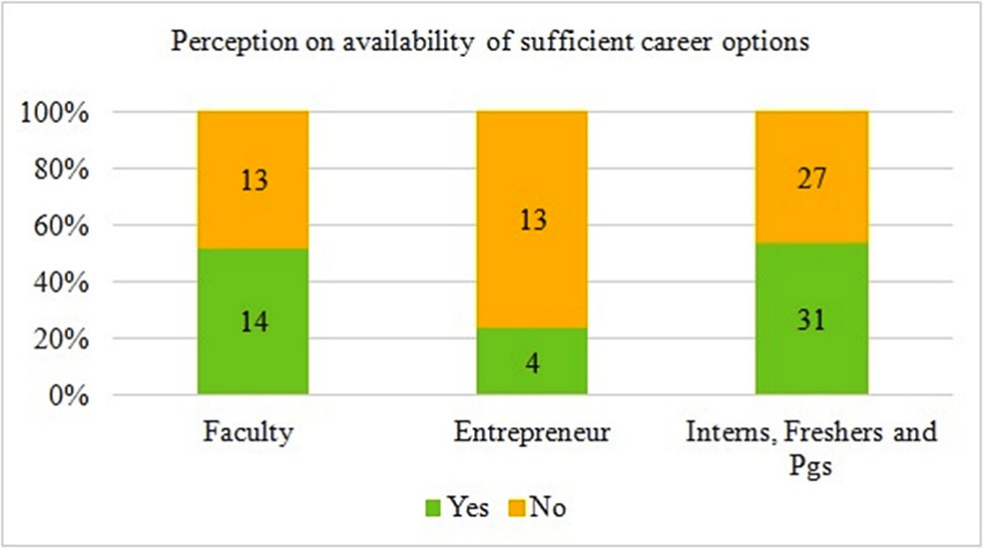
Perception of availability of sufficient career choices for Ayurveda graduates Total number of respondents = 102. PGs: postgraduates

Almost 50% of faculty (n=14) and student groups (n=31) felt that sufficient career options are available for Ayurveda graduates. However, only 23% of private practitioners and entrepreneurs (n=4) felt otherwise (Figure 3).

**Figure 4:**
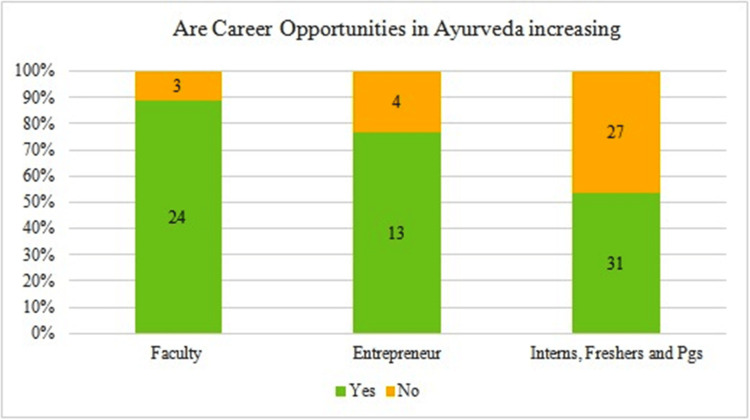
Perception of increased availability of career opportunities for Ayurveda graduates Total number of respondents = 102. PGs: postgraduates

The majority of stakeholders from all groups (faculty: n=24; entrepreneurs: n=13; interns: n=31) felt that career opportunities are increasing with globalization (Figure 4).

**Figure 5:**
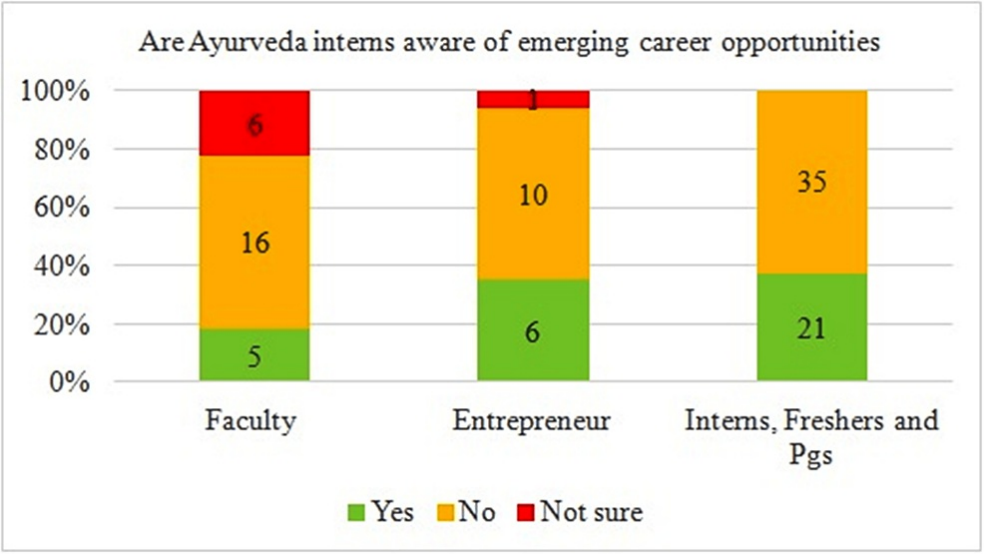
Perception of awareness of interns and fresh graduates regarding emerging career opportunities for Ayurveda graduates Total number of respondents = 102. PGs: postgraduates

Though many career opportunities are available, the majority of interns and fresh graduates (n=35) aren’t fully aware of the same (Figure 5).

**Figure 6:**
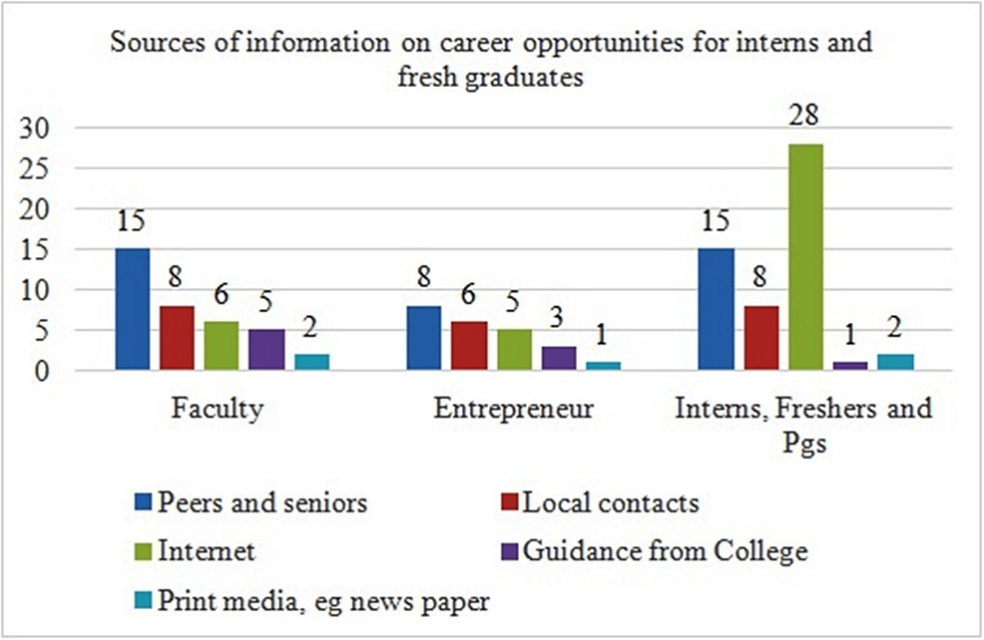
Sources of information on career opportunities for interns and fresh graduates Total number of respondents = 102. PGs: postgraduates

While faculty (n=23) and practitioners (n=14) felt that interns rely on peers and local contacts, for updated information, interns and fresh graduates themselves rely on the internet (n=28) over peers and local contacts (n=23). Guidance from colleges was minimally ranked among all (n=2) stakeholders (Figure 6).

Almost 75% of participants (n=77) were not aware of any forum for information on career opportunities. Over 98% (n=100) of respondents agreed with a need to have an orientation programme about career opportunities for Ayurveda interns and fresh graduates.

Over 76% (n=77) of participants commented that such training should be during the internship period. While 50% (n=51) of participants commented that Ayurveda colleges should provide such training, 32% (n=31) felt that universities should have a dedicated cell for the same. Eighteen per cent (n=20) of respondents commented that industry experts should be involved in such training programmes.

## Discussion

The objective of this Needs Assessment Survey was to find out whether there is a perceived need for introducing a career guidance module for Ayurveda interns and fresh graduates. Ayurveda graduates have the potential to be a suitable public health workforce due to their training, exposure, and interest, which can help replenish the country's public health workforce [4]. Internship is an important time in career decision-making. Junior doctors use their internship time to apply their knowledge and develop their clinical competency. Internship experiences are likely to influence the opinions of recently qualified graduates regarding their desired career paths [5]. Literature suggests that in the absence of informed decisions, students have expressed regret for their career choices [6].

The study brought various perspectives from various stakeholders regarding career guidance for Ayurveda interns and fresh graduates. Almost all survey participants agreed that there is an urgent need for career guidance in Ayurveda. This should not only focus on employment, but also help students in enhancing their communication skills, professional ethics and leadership skills along with clinical expertise.

Although today's Ayurvedic graduates have many career options to select from, they are not given any exposure to these options during their studies. This results in apathy towards the selection of any career apart from clinical practice. Interns in an Ayurvedic college show apathy towards research as a career option, highlighting the need to introduce research as a subject in the curriculum [7]. Short-term trainings have also shown to be effective [8].

The pursuit of a career in Ayurvedic research, Ayurvedic drug development, Ayurvedic manufacturing, Ayurvedic tourism, etc. lacks a clear career path. Most interns and recent graduates are unfamiliar with these uncharted career paths. The Health Universities, colleges and eminent experienced alumni of Ayurveda colleges can form an intensive network to guide and support students in making an appropriate choice of a career in Ayurveda. Career advice during undergraduate medical training is crucial, as most Indian medical students plan to prepare for PG exams, but receive minimal professional guidance from teachers [9].

By offering ongoing support and resources for professional growth, institutions can contribute to the continuous development of the Ayurvedic workforce [10]. Career guidance and counselling services significantly improve academic performance and graduate employability in students [11]. Improving employment guidance for college graduates can help them achieve full and satisfactory employment, addressing supply and demand conflicts and addressing competence and working unit requirements. Career counselling provided by health sciences institutions including Ayurveda colleges will help graduates reach good positions in an evolving and changing professional environment [12].

Strengths of the current study

A diverse group of stakeholder perspectives have been explored through this survey, which includes faculty, clinicians, industry entrepreneurs, residents, graduates and interns of Ayurveda. The questionnaire used was customised and validated as per the requirements of Ayurveda professionals. The snowball sampling technique helped ensure a wider audience.

Limitations of the current study and future recommendations

The potential limitations of the study include selection bias as the survey was distributed using WhatsApp. Future studies can focus on in-depth qualitative data to complement the questionnaire responses. The current findings are limited to the state of Maharashtra; further studies can explore perceptions from the broader Ayurvedic community from across the country.

## Conclusions

The provision of career guidance would definitely help students achieve their goal of becoming competent, skilful, and compassionate healthcare professionals who are well-suited for their chosen field of specialization. It would help undergraduates to develop their skills of decision-making required for coping with the needs of the real world and develop lifelong learning ambitions towards their field of choice. Introducing a career guidance module can enhance the overall educational experience for students in Ayurveda programs. By equipping them with the necessary tools and information to plan their careers effectively, educational institutions can better support their professional development and success.

There may be evolving market demands and trends within the field of Ayurveda that require a proactive approach to career planning and development. Understanding these market dynamics through a needs assessment can help tailor the career guidance module to align with current and future industry needs. Career guidance is not only beneficial for students but also for practising professionals looking to advance or transition within the field of Ayurveda. The findings of this survey can have long implications for Ayurveda education, practice, and policy. The findings suggest a need to incorporate career guidance formally into the curriculum, along with career counselling initiatives, and industry partnerships in the field of Ayurveda.

## Appendices

**Table 1:** Needs Assessment Survey for development of ‘Career Guidance Module’ for interns of Ayurveda Survey conducted after peer validation.

Needs Assessment Survey for development of ‘Career Guidance Module’ for interns of Ayurveda
Participant no:
(Note: Personal details are optional)
Name :
Age :
Sex :
Institute :
Designation :
Phone no :
Email :
Experience: Clinical :
Experience: Teaching :
Year of Graduation :
College of Graduation :
Year of Post-graduation :
College of Post-graduation :
Subject of Post-graduation :
The commonest career options available for graduates of Ayurveda are: [Please rate them in order of preference (from 1 to 8) according to you. You may specify and include Any Other option according to you.]
Ayurvedic Clinical practice
Join local hospitals as Resident medical officers (RMO)
Post-graduation
Academic
Administration
Research
Drug manufacturing
Any other (Please Specify)
Are sufficient career options available for Ayurveda graduates if they are not selected for M.D. course?
Yes
No
Do you feel that in today's era of globalization, opportunities for careers in Ayurveda are increasing?
Yes
No
Do you feel that interns of Ayurveda are aware of emerging new career opportunities in various fields?
Yes
No
Presently, how do you think the interns find out career opportunities for themselves? (Please rate in order from 1 to 5 according to your preference)
Through internet
Through local contacts
Through peers and seniors
Print media
Any other
Do you think there is a common platform for exchange of information between Interns and potential career opportunities?
Yes
No
Do you think there is a need for creating awareness about career options among interns of Ayurveda?
Yes
No
Do you think that, as Institutions of Ayurvedic Education, we can make our interns aware of various career opportunities?
Yes
No
Do you think there is a need for an orientation programme about career opportunities for interns of Ayurveda?
Yes
No
If yes, when should this orientation programme be conducted?
At the beginning of internship
During internship
At the end of internship
Any other?
Who should create such a module for orientation programme about career opportunities for interns of Ayurveda?
